# Microstructure, Tensile and Creep Properties of Ta_20_Nb_20_Hf_20_Zr_20_Ti_20_ High Entropy Alloy

**DOI:** 10.3390/ma10080883

**Published:** 2017-07-31

**Authors:** Natalya Larianovsky, Alexander Katz-Demyanetz, Eyal Eshed, Michael Regev

**Affiliations:** 1 Israel Institute of Metals, Foundry Laboratory, Technion-Israel Institute of Technology, Haifa 3200003, Israel; natalyal@trdf.technion.ac.il (N.L.); kalexand@techunix.technion.ac.il (A.K.-D.); eyale@trdf.technion.ac.il (E.E.); 2 Mechanical Engineering Department, ORT Braude College of Engineering, Karmiel 2161002, Israel

**Keywords:** high-entropy alloy, microstructure, X-ray diffraction, mechanical properties

## Abstract

This paper examines the microstructure and mechanical properties of Ta_20_Nb_20_Hf_20_Zr_20_Ti_20_. Two casting processes, namely, gravity casting and suction-assisted casting, were applied, both followed by Hot Isostatic Pressing (HIP). The aim of the current study was to investigate the creep and tensile properties of the material, since the literature review revealed no data whatsoever regarding these properties. The main findings are that the HIP process is responsible for the appearance of a Hexagonal Close Packed (HCP) phase that is dispersed differently in these two castings. The HIP process also led to a considerable increase in the mechanical properties of both materials under compression, with values found to be higher than those reported in the literature. Contrary to the compression properties, both materials were found to be highly brittle under tension, either during room temperature tension tests or creep tests conducted at 282 °C. Fractography yielded brittle fracture without any evidence of plastic deformation prior to fracture.

## 1. Introduction

Because modern jet engines require larger and larger parameters, improved creep properties are essential for the aerospace industry. The currently used Ni-based superalloys are reaching their limits, and since the beginning of the 21st century new alloys known as High Entropy Alloys (HEAs) have begun to look attractive [1,2]. HEAs can be regarded as solid solution alloys that contain at least five alloying elements in equal or near equal atomic percentages, and this large number of alloying elements results in maximizing the configurational entropy of the disordered solid solution. However, the microstructure of certain HEAs can include nano-precipitates, ordered solid-solution phases, disordered solid-solution phases, and even amorphous phases [1,2]. Among the various systems of alloying elements studied, the Ta_20_Nb_20_Hf_20_Zr_20_Ti_20_ alloy seems to be attractive due to its reduced density of 9.94 g/cm^3^ [2,3], few publications [4,5,6] deal with thr thermodynamic properties of certain compositions of the Ta-Nb-Hf-Zr-Ti.

As reported by Senkov et al. [2,3], the process of producing the Ta_20_Nb_20_Hf_20_Zr_20_Ti_20_ alloy consisted of vacuum arc melting followed by re-melting the material three times, five minutes each time, in order to achieve homogeneity. After that, the material underwent Hot Isostatic Pressing (HIP) at 1200 °C and 207 MPa for 1 h and finally underwent vacuum annealing at 1200 °C for 24 h.

According to Senkov et al. [2,3,7,8], after annealing at 1200 °C for 24 h the alloy was found to have a single-phase Body Centered Cubic (BCC) solid solution with a lattice parameter of 0.3404 nm, and its microstructure consisted of equiaxed, dendritic grains with an average size of 100–200 μm. Lin et al. [9] studied the microstructure of as-cast material (without HIP and annealing) and also reported the existence of dendrites and an interdendritic phase with slightly different chemical compositions. A single BCC structure was also reported by Maiti and Steurer [10], who studied Ta_18.5_Nb_20.8_Hf_23.5_Zr_25.2_Ti_12_ that was arc-melted and homogenized for four days at 1600 °C.

Senkov et al. [2] conducted compression tests on Ta_20_Nb_20_Hf_20_Zr_20_Ti_20_ at room temperature as well as at 600 °C, 800 °C, 1000 °C and 1200 °C under different strain rates and used Scanning Electron Microscopy (SEM) to investigate the microstructural changes during the deformation process and the fracture mechanisms. These researchers claimed that the above temperature range can be divided into three regions, each one characterized by different deformation behavior. According to Senkov et al. [2], at temperatures up to 600 °C twinning compensates for restricted dislocation mobility. At 800 °C Grain Boundary Sliding (GBS) is not yet supported by sufficient dislocation mobility and diffusion leads to cavitation at grain boundaries, while at 1000–1200 °C cavitation at grain boundaries disappears and Dynamic Recrystallization (DRX) occurs. DRX processes are assumed to be responsible for the rapid drop in the flow stress after yielding followed by a steady state flow.

The compression test data for Ta_20_Nb_20_Hf_20_Zr_20_Ti_20_ at room temperature include compression yield strength of 929 MPa [2] and 1073 MPa [9] together with fracture strain higher than 50% [9]. High temperature compression properties seem to be promising as well [2,8]. However, no data whatsoever have been published in reference to tensile properties and creep properties of as-cast Ta_20_Nb_20_Hf_20_Zr_20_Ti_20_ under tension. The current paper seeks to fill this gap by focusing on the tensile and creep properties of the Ta_20_Nb_20_Hf_20_Zr_20_Ti_20_ alloy together with its fracture mechanisms.

## 2. Results

**Figure 1:**
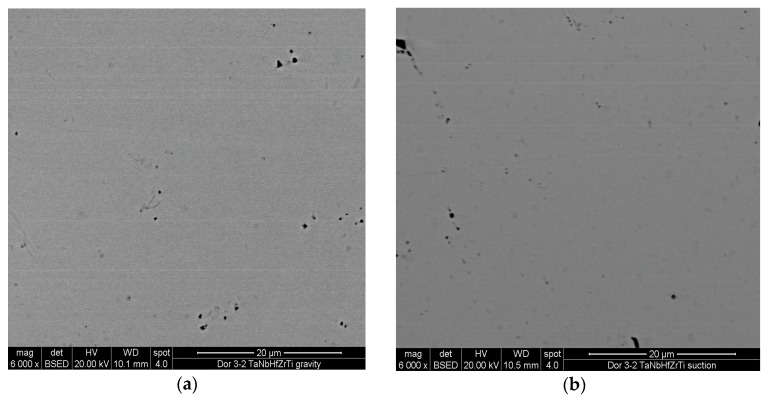

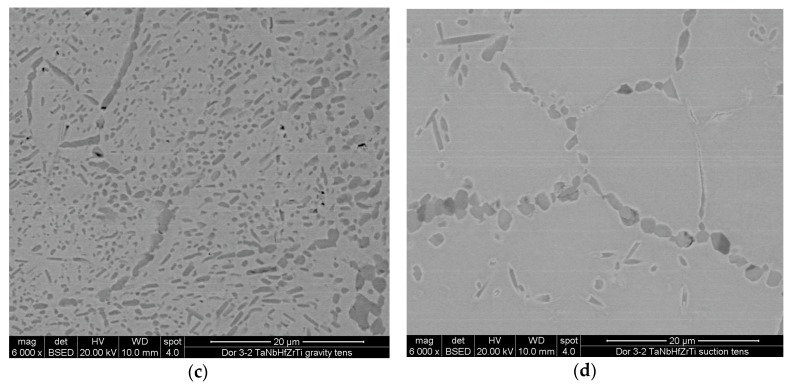
SEM images of the material: (**a**) gravity casting before HIP; (**b**) suction-assisted casting before HIP; (**c**) gravity casting after HIP; (**d**) suction-assisted casting after HIP.

Figure 1a depicts a back-scattered SEM image of the gravity-cast material prior to HIP, while Figure 1b shows the suction-assisted casting. Figure 1c,d depict the back-scattered SEM images of these two castings after HIP. Note that both materials were single phased before undergoing HIP, while a darker phase is discernible inside the bright matrix after HIP. Table 1 provides the respective Energy Dispersive X-ray Spectroscopy (EDS) analyses. It should be noted that the dark phase is evenly dispersed in the matrix of the gravity-assisted casting, while in the case of the suction-assisted casting it is concentrated mostly at the grain boundaries.

**Figure 2:**
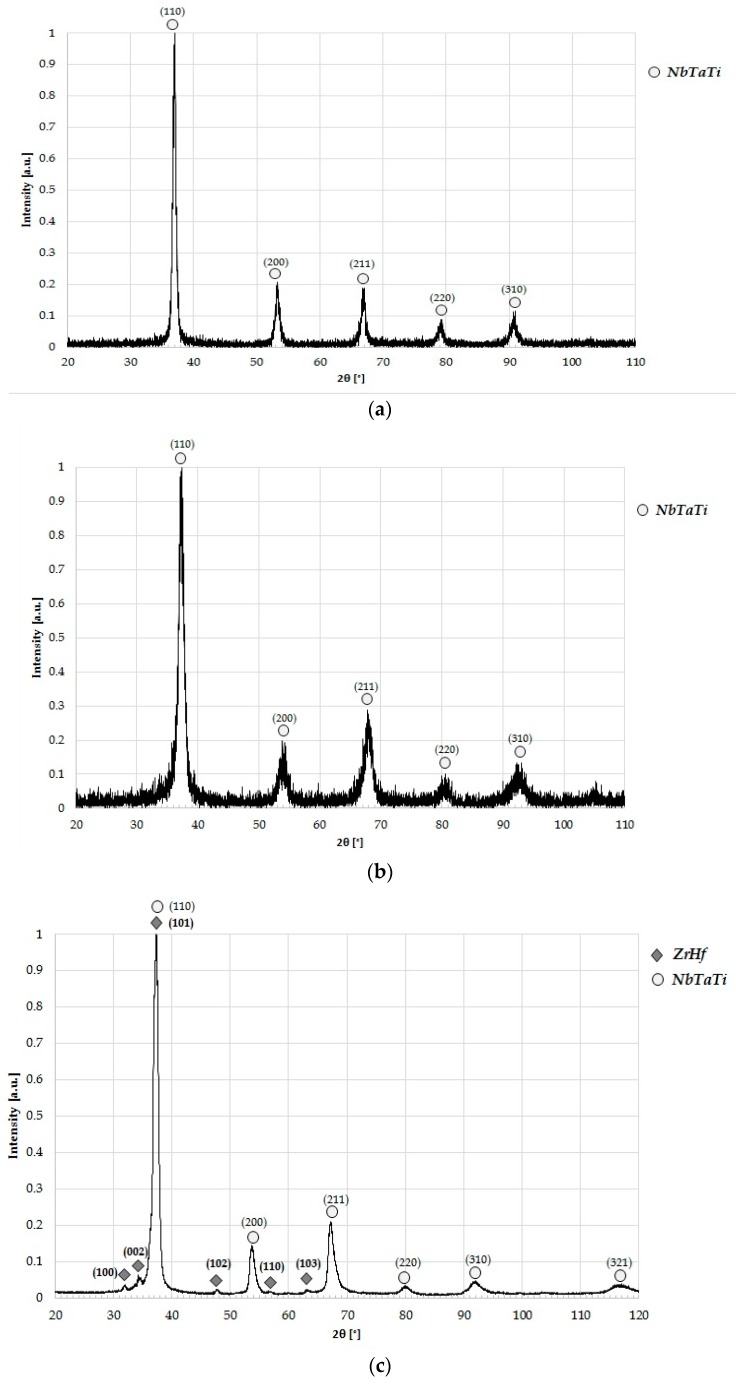

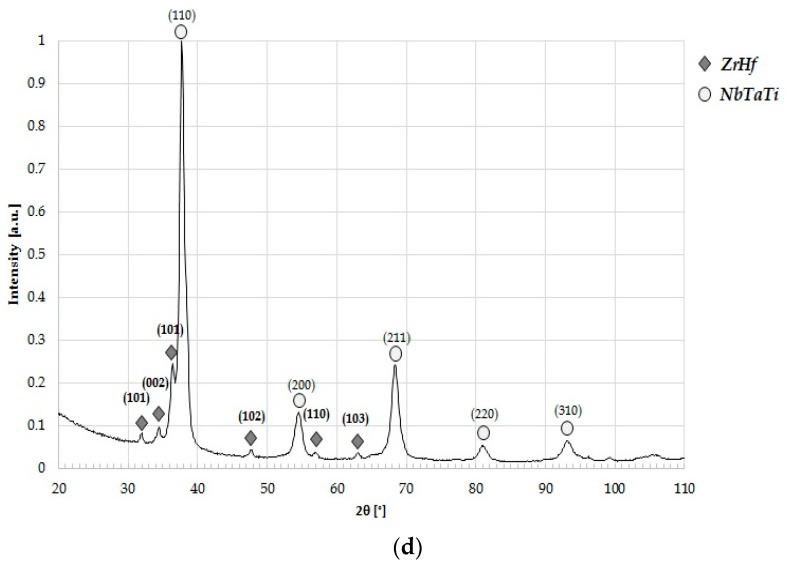
XRD spectra of HfNbTaTiZr alloy: (**a**) gravity casting before HIP; (**b**) suction-assisted casting before HIP; (**c**) gravity casting after HIP; (**d**) suction-assisted casting after HIP.

XRD spectra of both the gravity and the suction-assisted cast samples before HIP (see Figure 2a,b respectively) correspond to single-phase NbTaTi-based BCC material. The chemical compositions of the considered samples correspond to an overall equi-atomic material composition (see Table 1). Based on the above described SEM study, which indicates the presence of two major phases in the case of the HfNbTaTiZr alloy that has undergone HIP, each peak was associated with one of these two phases: a roughly equimolar solid solution of ZrHf and a solid solution of NbTaTi, again at roughly equimolar proportions. As ZrHf solid solution phase is comprised of HCP elements (at room temperature), it has an HCP structure and thus exhibits the characteristic X-ray diffraction pattern of other HCP phases. Contrary to the ZrHf, the NbTaTi solid solution phase is comprised of BCC elements and therefore has a characteristic X-ray diffraction pattern of other BCC-structured substances. Figure 2c shows the XRD spectra of the gravity-cast HfNbTaTiZr alloy and the HCP/BCC designation of each peak, while Figure 2d shows the spectrum of the suction-assisted cast, both after HIP.

Microhardness tests revealed the hardness of the material to be 323.5 ± 6.5 HV in the case of gravity casting and 330.2 ± 9.8 HV in the case of suction-assisted casting, both before HIP. For the material that underwent HIP, the hardness was found to be 437.2 ± 11.6 HV and 509.1 ± 13.5 HV for the gravity and suction-assisted castings respectively. Table 2 summarizes the tension and compression test results at room temperature.

The elongation to fracture under tension was below the machine’s detection limit and therefore could not be measured. The same was true for the differences between the yield stress and the ultimate tensile stress. Hence, the maximum stress measured is given in Table 2. Contrary to the tension tests, the yield stress could be easily detected in the case of the compression tests. Two creep tests at 982 °C were conducted on gravity-cast specimens, the first under a load of 200 MPa and the second under 120 MPa. Both failed after short periods of time of 15 and 90 min, respectively.

**Figure 3:**
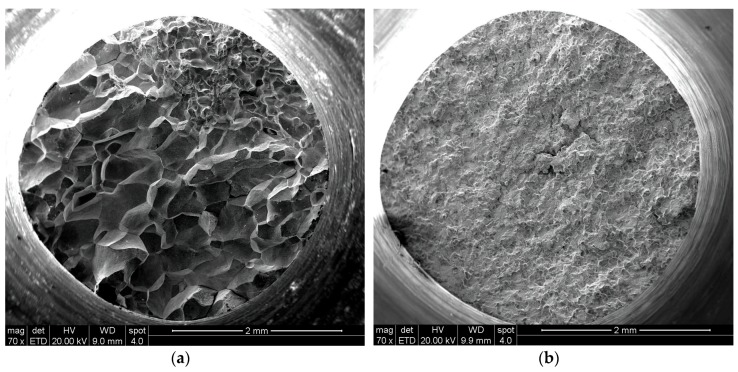
General view of the fracture surface of a tension specimen: (**a**) gravity casting; (**b**) suction-assisted casting.

Figure 3a,b provide a general view of the fracture surface of a gravity-cast and a suction-assisted-cast tension specimen, respectively. The figures show that the fracture is inter-crystalline in both cases, without any plastic deformation. The fracture of the suction-assisted casting is accompanied by intergranular cracking, which is most intensive at the center of the specimen. The grain size of the gravity casting varies significantly between the different regions of the fracture surface, while in the case of the suction-assisted casting the grain size is quite uniform.

**Figure 4:**
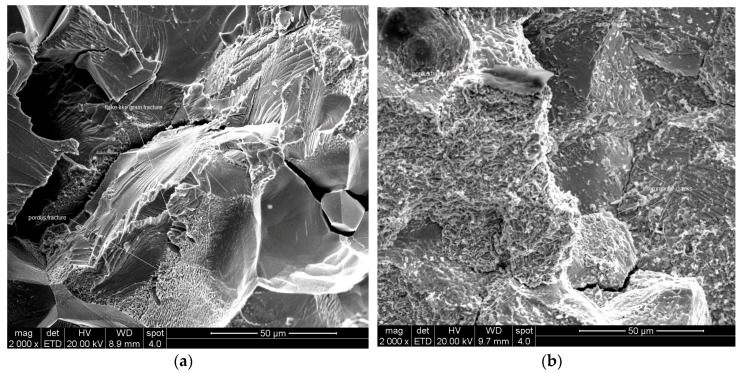
Fracture surface of: (**a**) gravity casting; (**b**) suction-assisted casting.

Figure 4 focuses on typical grains on the fracture surface of the above-mentioned tension specimens, showing that no evidence of plastic deformation is discernible in either case. In the case of the gravity cast, the observed grains are characterized by a flake-like surface structure together with some porous regions, while in the case of the suction-assisted cast, brittle fractured grains together with porous regions can be seen.

**Figure 5:**
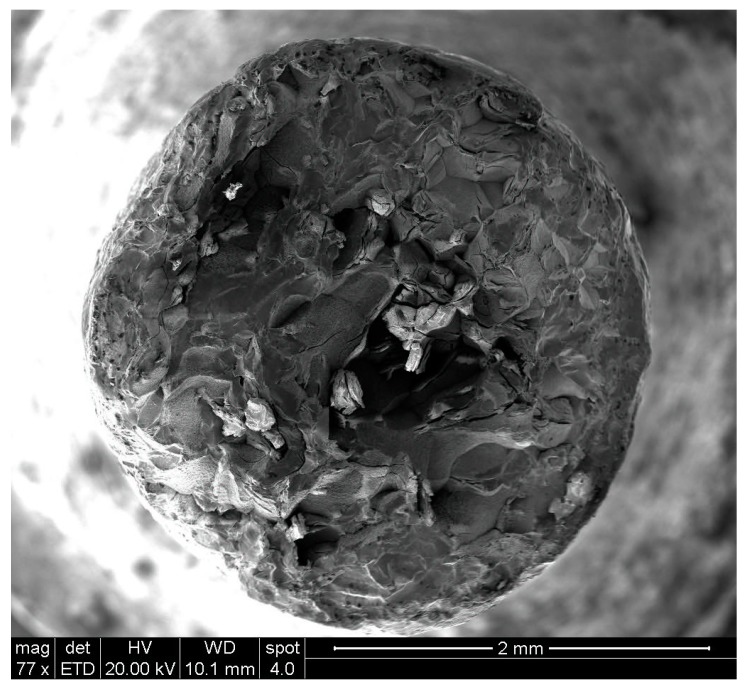
A general view of the fracture surface of a specimen that crept at 982 °C under 120 MPa.

**Figure 6:**
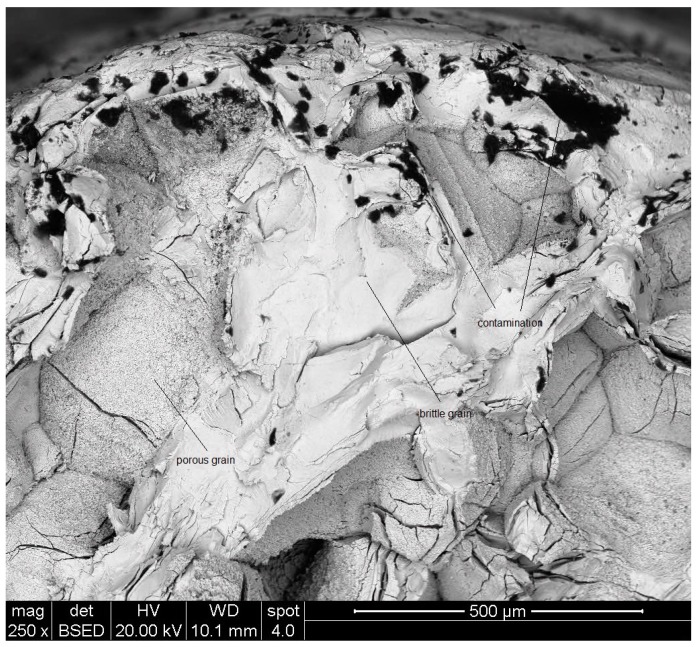
A BSE images of a selected region of the fracture surface shown in Figure 5.

Figure 5 depicts a SEM micrograph showing a general view of the fracture surface of a specimen that crept at 982 °C under 120 MPa. Figure 6 shows a Back Scattered Electron (BSE) image of a selected region of the fracture surface.

Figure 5 and Figure 6 show that the fracture is brittle. As revealed by EDS analysis, the surface is contaminated with oxidation products, mainly at the near-surface regions of the sample. Two main types of broken grains can be seen at the fracture surface: brittle cracked grains and porous grains.

## 3. Discussion

XRD and SEM analysis showed that both the gravity-cast specimen and the suction-assisted cast specimen had one single NbTaTi-based BCC phase, while both materials were composed of a NbTaTi-based BCC phase and a ZrHf HCP phase in their HIP’ed condition. Having analyzed their XRD spectrum, Senkov at al. [3] pointed to the existence of a BCC phase in both the cast and the HIP’ed conditions. Though these researchers reported on a small peak at 2θ = 24.9° indicating the presence of a hexagonal-like phase, they did not refer to the six additional HCP peaks detected in the current study in the case of HIP gravity casting: 2θ = 31.95°, 2θ = 34.28°, 2θ = 37.34°, 2θ = 47.66°, 2θ = 56.79° and 2θ = 63.23°. Nor did they refer to the following six peaks in the case of the suction-assisted casting: 2θ = 32°, 2θ = 34.3°, 2θ = 36.4°, 2θ = 47.5°, 2θ = 57° and 2θ = 62.6°. As stated earlier, identification of the chemical composition of the two phases of the material in its HIP’ed state is based on combining EDS analysis of the two detected phases with the XRD results. Senkov et al. referred to the microstructure of the Ta_20_Nb_20_Hf_20_Zr_20_Ti_20_ after undergoing HIP, claiming that only one BCC solid solution was discernible. However, they emphasized that it was not yet known whether the BCC phase is thermodynamically stable at Room Temperature (RT), or whether it is metastable and thus kinetically restricts formation of the low temperature HCP phase due to slow diffusivity [3]. The current study offers clear evidence of the existence of both a BCC and an HCP phase. It seems that the HIP process is responsible for the appearance of the HCP phase mentioned above. In addition, the hardness values of the gravity-cast material can be regarded as equal to those of the suction-assisted casting if we take into account the overlapping of the standard deviation of the two materials. As for the materials that underwent HIP, it is clear that the HIP process increased the hardness of both castings. Nevertheless, in the case of the materials that underwent HIP, the hardness of the suction-assisted casting was found to be greater than that of the gravity cast. The hardness increase after HIP can be related to the appearance of the secondary HCP phase and its influence on dislocation mobility, similarly to precipitation hardening. The difference between the suction-assisted cast and the gravity cast may be related to the distribution of the ZrHf-rich phase, which is evenly dispersed in the case of the gravity casting while concentrated mainly at the grain boundaries in the case of the suction-assisted casting. An explanation for the HIP process being responsible for the two-phased microstructure can be found when looking at the equilibrium phase diagrams of the elements comprising the alloy. Out of the five constituent elements, Nb and Ta are exclusively BCC elements, while the structure of Ti, Zr and Hf is HCP at low-to-moderate temperatures, undergoing allotropic transformation to BCC at high temperatures. Applying a pseudo-binary simplification and keeping in mind that Ti is the only HCP element, which can be easily stabilized as BCC at room temperature, it is sufficient to look at the elemental pairs Zr-Hf and Nb-Ta, as the HCP and BCC components of such a pseudo-binary system. According to the elemental equilibrium phase diagrams, upon cooling an alloy having a chemical composition close to the equimolar concentration ratio, either the high temperature BCC phase undergoes a eutectoid reaction or precipitation of the HCP component occurs. It is therefore reasonable to derive that when the HIP process of the HfNbTaTiZr alloy ends and the alloy is left to cool at a very slow rate inside the furnace the HCP phase forms out of the original BCC phase, either by a eutectoid reaction or by precipitation. In contrast, an HCP phase is not created in the case of the as-cast alloy due to the rapid cooling, resulting in a supercooled BCC phase. As mentioned earlier, the current study shows that an HCP ZrHf-rich phase appeared after HIP, however, further research is still required in order to confirm the proposed mechanism.

As stated earlier, to the best of the authors’ knowledge there are no published data whatsoever regarding tension properties or creep under tension. It should be noted that tensile properties were studied by Senkov et al. [7] but only after being 86.4% cold rolled and 86.4% cold rolled plus annealed at 800 °C and at 1000 °C. The mechanical properties of both castings under compression and under tension are markedly different, as can be seen from Table 2. σ_y_ under compression is almost as twice as high as the maximum stress under tension, while in the case of the compression tests conducted on the gravity casting, the results were relatively scattered. This higher degree of scattering may be related to the variation in grain size detected in the gravity casting as opposed to the uniform grain size of the suction-assisted casting. The values of σ_y_ under compression at room temperature are higher than those reported by Senkov et al.: 929 MPa [2], 1058 MPa [3] and 1073 MPa [9]. The elongation to fracture under tension at room temperature was beneath the machine’s detection limit and therefore could not be measured. Creep tests conducted at 982 °C led to premature failures. For the sake of comparison, according to MAR-M-247 material specifications for the aerospace industry [10,11], the time to rupture of the material should exceed 50 h under 200 MPa at 982 °C. These poor results under tension load lead to the conclusion that the material is extremely brittle and therefore cannot withstand any tension stress applied either at room temperature or at high temperatures. Senkov et al. [7] reported that the 86.4% cold-rolled material showed true tensile strength of 1295 MPa and tensile ductility of 4.7%, however, cold rolling resulted in an extensive grain elongation, formation of deformation bands within the grains, and development of crystallographic textures that depended on the rolling reduction. Annealing lead to recrystallization and to the formation of fine second-phase precipitates which could not be characterized by them [7]. These results, in turn, lead to the conclusion that both the microstructure and the mechanical properties of the HIP’ed material and those of the HIP’ed plus cold rolled material are markedly different.

As stated earlier, fractography studies of broken tension test specimens in the case of the gravity casting revealed significant variations in grain size between the different regions of the fracture surface, as opposed to the uniformity of the grain size in the suction-assisted casting. In turn, this may point to casting inhomogeneity in the case of gravity casting, so that suction-assisted casting is preferable. The lack of any evidence of plastic deformation in both gravity and suction-assisted casting is in line with the non-detectable elongations to fracture. The high concentration of oxides beneath the surface detected in the broken creep specimens may be due to preexisting surface cracks through which the oxidation process occurred. Nonetheless, the existence of such open surface cracks, their propagation process and their influence on the poor creep properties of the material under tension should be further investigated.

## 4. Materials and Methods

The Ta-Nb-Hf-Zr-Ti alloy in the form of buttons was prepared by vacuum arc melting of the nominal mixtures of the corresponding elements. The degrees of purity of Ta, Nb, Hf, Zr, and Ti were 99.9%, 99.9%, 99.7%, 99% and 99.7%, respectively. Melting was conducted in a high-purity argon atmosphere. High-purity molten titanium was used as a getter for residual oxygen, nitrogen, and hydrogen. In order to achieve homogeneous distribution of the elements in the alloys, the buttons were turned upside down and re-melted four times. The buttons were approximately 13 mm thick and 35 mm in diameter and had a shiny surface. Following this stage, the alloys were cast into bars of 9 mm in diameter and 85 mm in length. One group of specimens was left to solidify inside the mold after casting. Namely, it underwent a process of gravity casting. The other group was cast by applying suction by means of a vacuum pump. The last stage was Hot Isostatic Pressing (HIP), HIP is a common practice, involving high temperatures and isostatic pressure and it is used for closing internal porosity [12]. HIP was applied in other studies as well as reported [2,3,7,8]. In the current study, the bars underwent HIP at 1230 °C and 152 MPa for 4 h. The bars were then radiographically examined in order to eliminate the existence of porosity and other defects that may lead to premature failure under tension. X-ray Diffraction (XRD) tests were performed using a Stationary Rigaku Smart Lab diffractometer (Tokyo, Japan) equipped with a Cu tube (λ_Kα_ = 1.5406 Å). An FEI Inspect SEM (Brno, Czech Republic) equipped with an Oxford Energy Dispersive X-ray Spectroscopy (EDS) system was used to analyze the microstructure and the chemical composition of the alloy and its phases as well as the fracture surface. Vickers hardness measurements were conducted using a Seiki Matsuzawa microhardness tester (Tokyo, Japan) under a load of 500 gf. Ten measurements, each lasting 15 s, were taken from the gravity and suction-assisted casts in both states—before and after HIP. Tension, compression and creep specimens were prepared from the HIP’ed bars by machining. Room temperature tension tests and creep tests were conducted on similar dog-bone specimens, while compression tests were conducted on cylindrical specimens having a diameter of 8 mm. Both creep and tension specimens were manually polished prior to being tested in order to eliminate machining scratches.

**Figure 7:**
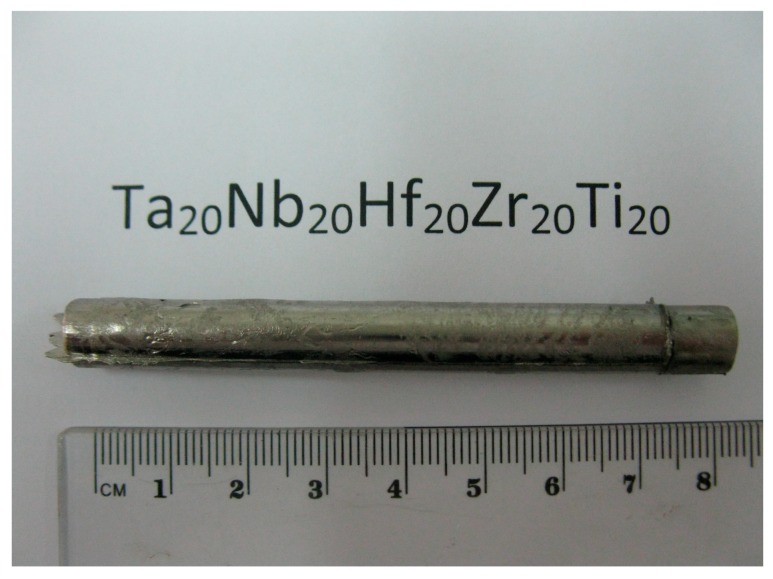
An as-cast bar.

**Figure 8:**
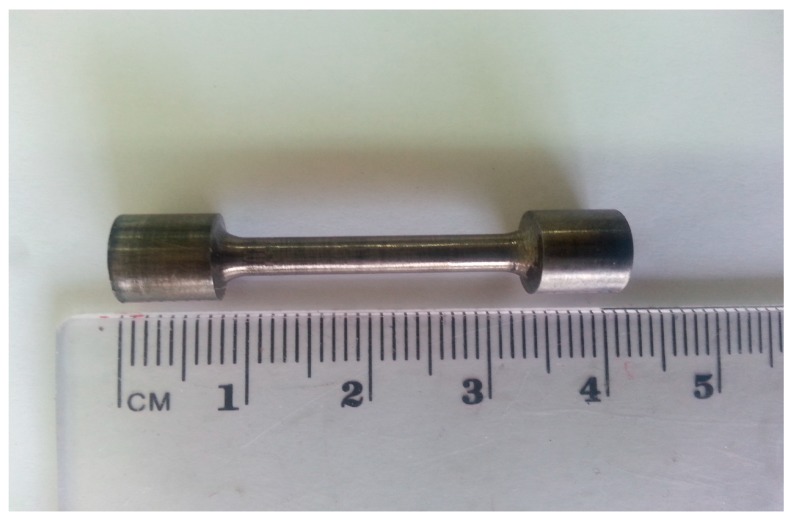
A tension/creep specimen.

Figure 7 shows the cast bar, while Figure 8 depicts a tension/creep specimen. The fracture surfaces of the broken tension and creep specimens were examined by SEM.

Table 3 shows the alloy composition in its as-cast state.

## 5. Conclusions

- The microstructure and mechanical properties of gravity-cast and suction-assisted cast Ta_20_Nb_20_Hf_20_Zr_20_Ti_20_ were studied.
- The HIP process, applied to both castings, was found to be responsible for the appearance of an HCP phase in addition to the preexisting BCC stage. The HIP process also increased the hardness of both castings, and in the case of the suction-assisted castings led to a larger increase.
- The HCP phase that appeared after HIP is evenly dispersed in the matrix of the gravity-assisted casting, while it is concentrated mostly at the grain boundaries in the case of the suction-assisted casting.
- Compression strength values measured for both gravity casting and suction-assisted casting in the current study are higher than those reported in the literature.
- Creep and tension test results as well as fractography showed that both materials are extremely brittle under tension.
- A fractography study of the broken tension test specimens revealed significant variations in grain size between the different regions of the fracture surface in the case of the gravity casting, as compared to the uniformity of the grain size in the suction-assisted casting.
- High concentration of oxides observed beneath the surface of the broken creep specimens may point to preexisting surface cracks through which the oxidation process occurred. Nevertheless, further research is still required in order to understand the failure mechanism.

## Figures and Tables

**Table 1:** Energy Dispersive X-Ray Spectroscopy (EDS) results (at %).

Sample	Phase	Ti	Zr	Nb	Hf	Ta
Gravity casting before HIP	overall composition	19.11	20.66	21.15	19.85	19.24
Suction-assisted casting before HIP	overall composition	19.13	21.42	22.12	19.46	17.86
Gravity casting after HIP	overall composition	19.10	21.36	21.53	18.82	19.18
	matrix	20.30	21.42	27.35	10.78	20.15
	darker phase	10.88	48.79	4.00	33.26	3.07
Suction-assisted casting after HIP	overall composition	20.62	22.45	25.73	13.31	17.90
	matrix	20.13	21.58	27.70	10.92	19.67
	darker phase	8.52	57.38	3.12	28.51	2.47

**Table 2:** Tension and compression test results.

Test	Process	σ_y_ (Mpa)	σ_max_ (Mpa)
Tension	Gravity casting		669
	Gravity casting		749
	Suction-assisted casting		694
	Suction-assisted casting		708
	Suction-assisted casting		678
Compression	Gravity casting	1380	
	Gravity casting	1350	
	Gravity casting	1749	
	Suction-assisted casting	1725	
	Suction-assisted casting	1684	
	Suction-assisted casting	1888	

**Table 3:** Chemical composition of the as-cast alloy (at %).

Alloy	Ta	Nb	Hf	Zr	Ti
TaNbHfZrTi	20.4	20.1	20.6	20.7	18.2
